# Possible Diclofenac-Related Fulminant Toxic Epidermal Necrolysis: A Case Report

**DOI:** 10.7759/cureus.110928

**Published:** 2026-06-15

**Authors:** Natalia Quintero Serrano, Olga Lucía Melo Trujillo

**Affiliations:** 1 Clinical Toxicology, Fundación Universitaria de Ciencias de la Salud (FUCS), Bogotá, COL

**Keywords:** adverse drug reaction, diclofenac, nonsteroidal anti-inflammatory agents, scorten, toxic epidermal necrolysis

## Abstract

Toxic epidermal necrolysis (TEN) is a rare but life-threatening cutaneous adverse effect characterized by extensive epidermal detachment and systemic inflammation. Though anticonvulsants, sulfonamides, and allopurinol are most frequently associated with TEN, nonsteroidal anti-inflammatory drugs (NSAIDs) such as diclofenac have been involved in very few case reports. This case study presents a 76-year-old man with tophaceous gout who, after a self-administered single dose of 75 mg intramuscular diclofenac, developed fulminant TEN. At the time of hospital admission, the patient's skin showed progressively developing purpuric and bullous lesions covering 40.5% of the total body surface area, a positive Nikolsky sign, severe mucosal involvement, metabolic acidosis, acute kidney failure, thrombocytopenia, and multiorgan failure. Despite aggressive supportive care management, the patient died shortly after hospital admission. Based on the SCORe of Toxic Epidermal Necrosis (SCORTEN) severity score of 5, the patient was classified as having a very high risk of mortality, corresponding to an estimated mortality rate of approximately 85%. Using the Naranjo algorithm for evaluating the causal relationship between a drug therapy and a possible side effect, we obtained a score of 2, which points to a possible side effect, while the re-exposure to febuxostat, etoricoxib, and pregabalin was an alternate pharmacological variable during the course of the adverse event. This case highlights the potential for rapidly progressive and fatal TEN following recent NSAID exposure and underscores the importance of early recognition, prompt withdrawal of suspected medications, and careful causality assessment in patients receiving multiple drugs.

## Introduction

Toxic epidermal necrolysis (TEN) is the most serious among the range of severe cutaneous adverse reactions, being the endpoint of the progression from milder forms such as Stevens-Johnson syndrome (SJS) and SJS-TEN overlap. Based on the criterion of epidermal necrosis affecting more than 30% of the total body surface area, this condition has been associated with a 20-35% in-hospital mortality rate, increasing drastically as a function of patients' age and higher SCORe of Toxic Epidermal Necrosis (SCORTEN) scores [1]. In terms of its pathology, TEN involves extensive death of epidermal keratinocytes due to apoptosis induced by T lymphocytes, natural killer cells, and several soluble molecules like granulysin, Fas ligand, perforin, and granzyme B, leading to full epidermal necrosis [2,3].

Regarding pharmaceutical factors that contribute to the development of TEN, aromatic anticonvulsants (carbamazepine, phenytoin, and lamotrigine), antimicrobials (sulfonamides and beta-lactams), allopurinol, and nevirapine are among the most common ones [4]. Nonsteroidal anti-inflammatory drugs (NSAIDs), comprising the acetic acid derivative diclofenac, are less commonly implicated drugs in the literature as culprits, yet have been described in isolated case reports and pharmacovigilance registries in association with the induction of SJS and TEN [5,6]. Diclofenac is one of the most prescribed painkillers and anti-inflammatory drugs, being available in oral, intravenous, and transdermal forms.

This report presents an illustrative case of a fatal episode of fulminant TEN occurring in an elderly male after the administration of a single dose of intramuscular diclofenac, in association with concurrent re-exposure to the NSAID etoricoxib, the xanthine oxidase inhibitor febuxostat, and the neuroleptic pregabalin. This report aims to provide further evidence of the potential role of NSAIDs in the pathogenesis of fatal episodes of TEN in association with the toxicological mechanism underlying this severe immune-mediated reaction.

## Case presentation

A review of the patient's medical history indicated that the patient was a 76-year-old man suffering from tophaceous gout, following a previous surgery for the amputation of the left foot due to gout-related tophi. The patient came to the emergency department on day 5, complaining of an acute episode of systemic decompensation within five days. As reported by the patient in the course of the clinical consultation, his chronic drug regimen had comprised prednisolone 50 mg once daily, febuxostat 40 mg twice daily, etoricoxib 60 mg once daily, and esomeprazole 40 mg once daily. He had ceased all these drugs around two months back.

Specifically, he had started again on prednisolone, febuxostat, and etoricoxib at their previously prescribed doses on day 1, after experiencing a flare of his gout in his right hand. The last use of these drugs was the night before, day 4 (evening before admission). In addition, pregabalin use had begun two days before hospitalization at an unknown dosage. Additionally, he had one dose of self-medicated diclofenac 75 mg injected intramuscularly into his left gluteal muscle on day 2.

Twenty-four hours after the administration of diclofenac injection on day 3, the patient experienced severe, stabbing pain affecting the left buttock that radiated to his legs and the abdominal wall, along with the development of progressive edema and changes in skin color, including mottling. Over the next 48 hours, the condition progressed, with bullae appearing in addition to violaceous-purple purpuric macular lesions that spread from the trunk to the extremities. The presentation of symptoms was already in its fulminant phase when the patient came into the hospital on day 5.

During triage on day 5, at the time of emergency department presentation, the patient was assessed as being Triage II with an emergency diagnosis of shock (International Classification of Diseases, 10th Revision (ICD-10): R57.8). On examination, his vital signs were as follows: blood pressure 149/81 mmHg, mean arterial pressure 103 mmHg, heart rate 119 bpm, respiratory rate 17/min, oxygen saturation 80% on nasal oxygen (fraction of inspired oxygen (FiO₂): 28%), temperature 36.7°C, pain rating 7/10, weight 66 kg, height 168 cm, and BMI 23.3 kg/m^2^.

**Figure 1:**
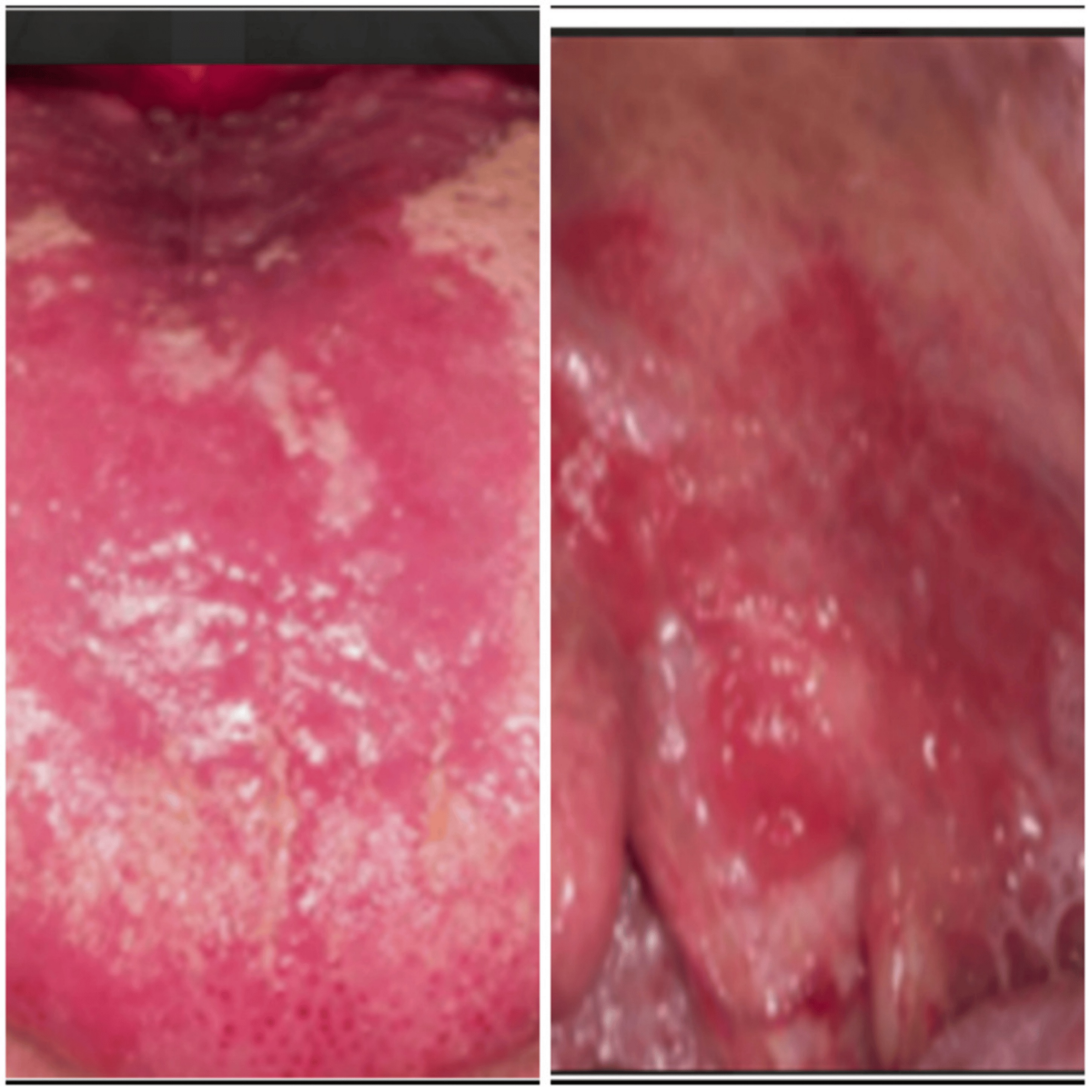
Oral and oropharyngeal mucosal involvement characterized by erythematous glossitis with patchy superficial depapillation and erosions of the tongue, accompanied by diffuse oropharyngeal erythema, erosive ulceration, and sloughing of the soft palate and peritonsillar region

The Glasgow Coma Scale score was 15/15 with no focal neurological abnormality and no meningeal signs. Cutis marmorata and hypothermia were observed on general examination. Mucous membranes were dry, with hyperemia and focal peeling. Further evaluation by a dedicated oral examination confirmed that the patient had erythematous glossitis with patchy areas of superficial depapillation and erosion of the dorsum of the tongue and the soft palate and peritonsillar areas showed diffuse erythema with erosive ulcers, with sloughing (Figure 1). Cardiorespiratory examination was stable. Abdominal examination was non-contributory. Placement of an orogastric tube with escalation of care revealed approximately 100 cc of coffee-ground material.

**Figure 2:**
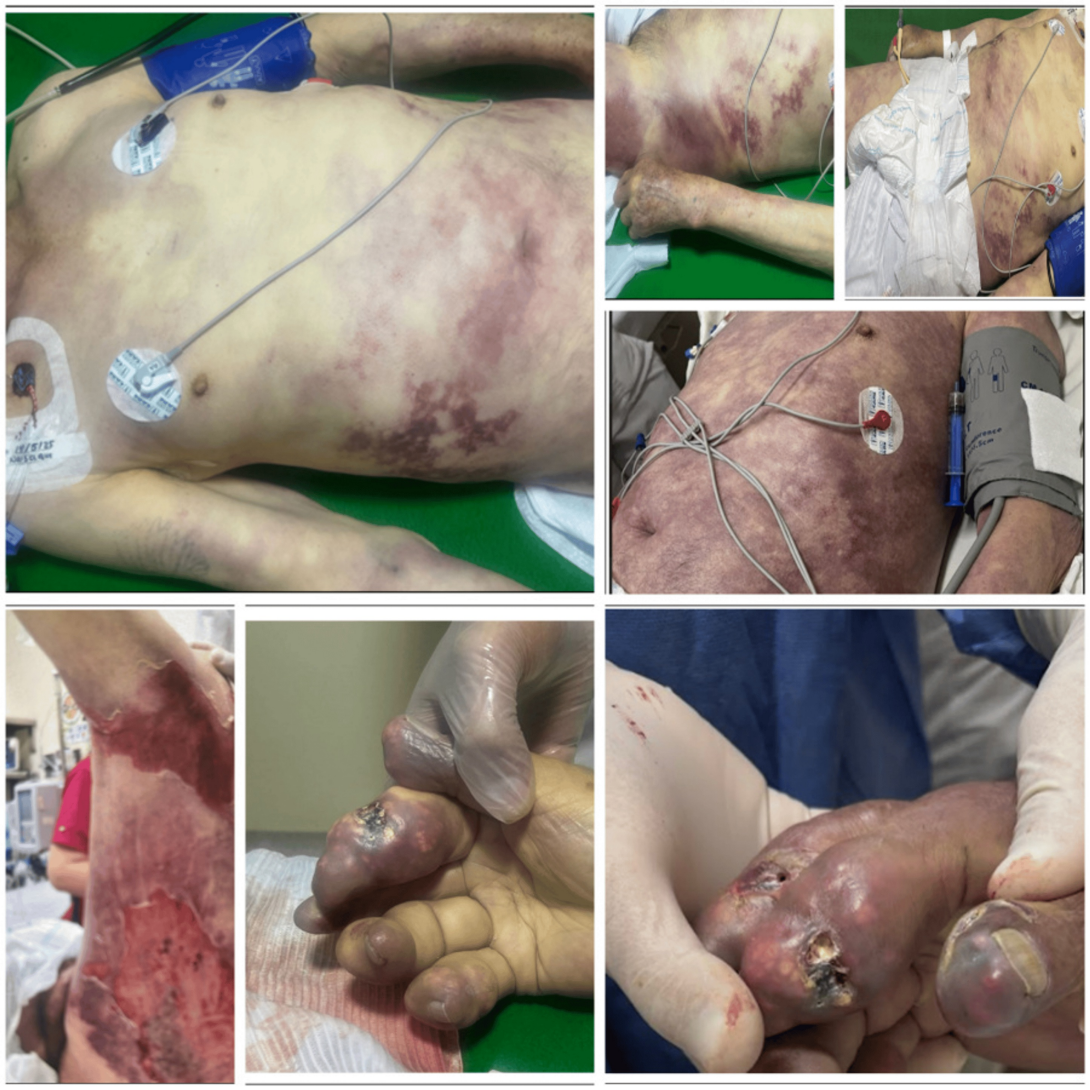
Extensive mucocutaneous involvement characterized by widespread purpuric and violaceous macules, patches, and confluent discoloration affecting the thoracic, abdominal, truncal, upper extremity, and lower extremity regions, with extensive epidermolysis, epidermal detachment, and large denuded erosive areas. The right hand demonstrates advanced tophaceous gout involving the second digit, with associated soft tissue necrosis, tissue loss, and necrotic changes

Physical findings of cutaneous involvement were diagnostic for TEN. Large areas of extensive purpuric lesions with some epidermal detachments were identified on the thorax and abdomen. Purpuric macules with epidermolysis were observed on the lateral trunk. In addition, purpuric and dusky violaceous macules and patches affected the thoraco-abdominal region and proximal lower extremity. Violaceous patches with de-epithelialization were evident on the left thigh, specifically a 30×10 cm area on the posterolateral side of the left thigh, along with violaceous patches with de-epithelialization on the lateral aspect of the left knee, with a size of 10×15 cm. In the lower extremities, extensive erosions, denuded areas, violaceous discoloration, and residual detachable epidermis on the periphery of the lesion were seen. Gangrene with loss of tissue was seen on the second digit of the right hand (Figure 2). Livedo reticularis was observed in the anterior and posterior trunks. A positive Nikolsky's sign was observed. No evidence of dehiscence or ischemia was noted in the left foot amputation stump. Pulses were palpable bilaterally. Total body surface area involved with lesions was 40.5%.

Laboratory tests showed that there was significant impairment to the body's physiology (Table 1). The white blood cell count was 6.40×10³/µL, with marked neutrophilia (87.9%). Absolute lymphocyte count showed 300/µL, thus indicating the presence of lymphopenia. The hemoglobin level was found to be 16.5 g/dL. Other hematological factors included a hematocrit of 49.7% and a platelet count of 91,000/µL. Renal dysfunction was severe, as evidenced by the presence of serum creatinine of 3.71 mg/dL and blood urea nitrogen of 80.43 mg/dL. The levels of glucose and sodium were noted at 123 mg/dL and 142 mmol/L, respectively. Laboratory results for coagulation included a prothrombin time of 16.7 s, an international normalized ratio (INR) of 1.2, and a partial thromboplastin time of 39.5 s. Arterial blood gas analysis revealed severe metabolic acidosis with pH 7.10, partial pressure of arterial carbon dioxide (PaCO₂) 18.5 mmHg, bicarbonate 6.9 mmol/L, and base excess -18.8 mmol/L. A terminal arterial blood gas obtained during resuscitation documented the following: pH 6.97, PaCO₂ 34 mmHg, partial pressure of arterial oxygen (PaO₂) 367 mmHg, bicarbonate 7.6 mmol/L, base excess -23.4 mmol/L, and lactate 6.21 mmol/L.

**Table 1:** Laboratory investigations at presentation Reference ranges may vary slightly between laboratories. *Elevated PaO₂ reflects measurement during advanced oxygen supplementation and mechanical ventilation.

Parameter	Patient value	Normal reference range
White blood cell count (WBC)	6.40×10³/µL	4.0-11.0×10³/µL
Neutrophils	87.9%	40-75%
Absolute lymphocyte count	300/µL	1000-4800/µL
Hemoglobin	16.5 g/dL	13.5-17.5 g/dL
Hematocrit	49.7%	41-53%
Platelet count	91×10³/µL	150–450×10³/µL
Serum creatinine	3.71 mg/dL	0.70-1.30 mg/dL
Blood urea nitrogen (BUN)	80.43 mg/dL	7-20 mg/dL
Serum glucose	123 mg/dL	70-110 mg/dL
Sodium	142 mmol/L	135-145 mmol/L
Prothrombin time (PT)	16.7 s	11-13.5 s
International normalized ratio (INR)	1.2	0.8-1.2
Partial thromboplastin time (PTT)	39.5 s	25-35 s
Arterial pH	7.10	7.35-7.45
PaCO₂	18.5 mmHg	35-45 mmHg
Bicarbonate (HCO₃⁻)	6.9 mmol/L	22-28 mmol/L
Base excess	-18.8 mmol/L	-2 to +2 mmol/L
Terminal arterial pH	6.97	7.35-7.45
Terminal PaCO₂	34 mmHg	35-45 mmHg
Terminal PaO₂	367 mmHg*	80-100 mmHg
Terminal bicarbonate (HCO₃⁻)	7.6 mmol/L	22-28 mmol/L
Terminal base excess	-23.4 mmol/L	-2 to +2 mmol/L
Lactate	6.21 mmol/L	0.5-2.2 mmol/L

The portable chest X-ray performed at 18:25 hours showed bilateral apical consolidation, bibasilar alveolar infiltrate, evidence of magnification of the heart, proper placement of the endotracheal tube (11 mm from the carina), a right subclavian catheter tip within the internal jugular vein, and no pleural effusion or pneumothorax. It was suggestive of pulmonary edema or pneumonitis due to inflammation.

The SCORTEN score for severity of illness in TEN was 5, indicating a predicted in-hospital mortality of about 85% (Table 2). The total number of points was awarded for the following criteria: age over 40 years (76 years old), heart rate over 120/min (highest recorded heart rate 140 bpm), percentage of epidermal detachment over 10% (40.5%), blood urea nitrogen over 28 mg/dL (80.43 mg/dL), and bicarbonate under 20 mmol/L (6.9 mmol/L). Serum glucose over 252 mg/dL (serum glucose 123 mg/dL) and malignancy (no malignancy present) did not award any points.

**Table 2:** SCORTEN severity-of-illness score: parameter breakdown and predicted mortality A SCORTEN score of 5 corresponds to an estimated in-hospital mortality of approximately 85%. SCORTEN: SCORe of Toxic Epidermal Necrosis; BSA: body surface area; BUN: blood urea nitrogen; HCO₃⁻: bicarbonate; bpm: beats per minute

SCORTEN parameter	Patient value	Score assigned
Age >40 years	76 years	+1
Heart rate >120 bpm	140 bpm	+1
Active malignancy	None	0
Epidermal detachment >10% BSA	40.5% BSA	+1
Serum BUN >28 mg/dL	80.43 mg/dL	+1
Serum HCO3 <20 mmol/L	6.9 mmol/L	+1
Serum glucose >252 mg/dL	123 mg/dL	0
Total SCORTEN score	-	5 (predicted mortality: ~85%)

The following list includes the differential diagnoses entertained at the point of presentation: necrotizing fasciitis, toxic shock syndrome, necrotizing soft tissue infection of the left lower extremity, and severe sepsis with purpura fulminans. As the dermatological and other specialist consultations were completed, each of the differential diagnoses was ruled out until the definitive diagnosis of TEN (ICD-10: L51.2-TEN/Lyell) was established by the clinical consensus of SCORTEN 5 with potential NSAIDs as causality.

Immediate emergency management was commenced following admission. Fluid resuscitation was started using lactated Ringer's fluid, with an initial dose of 500 ml, followed by maintenance fluid therapy, which increased to 2000 ml upon the worsening of the clinical condition. An empirical dose of 1 gram of vancomycin administered intravenously every 12 hours was commenced early on in light of the possibility of toxic shock syndrome and necrotizing soft tissue infection. Due to the clinical decline of the patient, intubation was performed with fentanyl 50 mcg, propofol 50 mg, and rocuronium 50 mg. The use of mechanical ventilation was then continued with the insertion of an 18-French orogastric tube. Central venous cannulation was performed through the right subclavian vein due to difficulty accessing peripheral veins. Vasopressors were commenced in the form of norepinephrine, starting at 0.3 mcg/kg/min.

After the clinical diagnosis of TEN due to the existence of epidermal detachment, mucosal involvement, and positive Nikolsky's sign had become the choice, suitable management was initiated. Histological diagnosis could not be made due to the rapidly deteriorating condition of the patient; however, the following differential diagnoses were entertained: purpura fulminans, toxic shock syndrome, septic vasculopathy, necrotizing soft tissue infection, and drug reaction with eosinophilia and systemic symptoms (DRESS) syndrome. The administration of methylprednisolone 52 mg via the intravenous route was carried out. Cyclosporine was withheld owing to the presence of acute renal damage. Petrolatum-impregnated gauze was used for wound dressings on erosions on the left lower limb, which were then dressed with elastic bandages. Albendazole 400 mg orally in a single dosage was provided before the commencement of corticosteroids as a preventive measure against parasitic infection according to institutional guidelines. Omeprazole 80 mg was provided intravenously in the form of a loading dose, and subsequently, 40 mg intravenously every 12 hours was given as gastroprotection. Intravenous immunoglobulins were not provided. Ocular protection measures were started.

The condition became severe and nonresponsive. During treatment in the resuscitation room with advanced cardiorespiratory therapy, monitoring in bed showed peaks in the T waves, indicating hyperkalemia, which eventually developed into asystole and no peripheral pulses. Based on the possibility of cardiac arrest due to hyperkalemia, the following treatments were given according to the guidelines for advanced cardiac life support: one ampule of calcium gluconate, one ampule of magnesium sulphate, and 1 mg of intravenous epinephrine, together with continuous cardiopulmonary resuscitation (CPR) and pulse assessment every three minutes. Following the 12-minute resuscitation attempt, the patient was pronounced dead on day 5, following unsuccessful resuscitation efforts, because of the inability to restore spontaneous circulation. The patient survived for about seven hours after his admission to the emergency department until his pronouncement of death. Given the temporal relationship between diclofenac administration and the onset of TEN, a causality assessment was performed using the Naranjo Adverse Drug Reaction Probability Scale to evaluate the likelihood of a drug-related adverse reaction (Table 3).

**Table 3:** Naranjo Adverse Drug Reaction Probability Scale: completed for diclofenac and TEN A total Naranjo score of 2 indicates a "possible" adverse drug reaction. ADR: adverse drug reaction; TEN: toxic epidermal necrolysis

Naranjo question	Yes	No	Score
1. Previous conclusive reports on this reaction?	+1	0	0 (unknown)
2. Did the ADR appear after drug administration?	+2	-1	+2
3. Did ADR improve after drug discontinuation?	+1	0	0 (single dose)
4. Did ADR recur on rechallenge?	+2	-1	0 (not done)
5. Were there alternative causes for the reaction?	-1	+2	-1 (yes)
6. Did ADR reappear on placebo?	-1	+1	0 (not done)
7. Was drug detected in toxic concentrations?	+1	0	0 (not measured)
8. Was reaction dose-dependent?	+1	0	0
9. Prior similar reaction to this drug class?	+1	0	0 (none)
10. ADR confirmed by objective evidence?	+1	0	+1 (clinical)
Total Naranjo score	-	-	2 (possible ADR)

## Discussion

**Figure 3:**
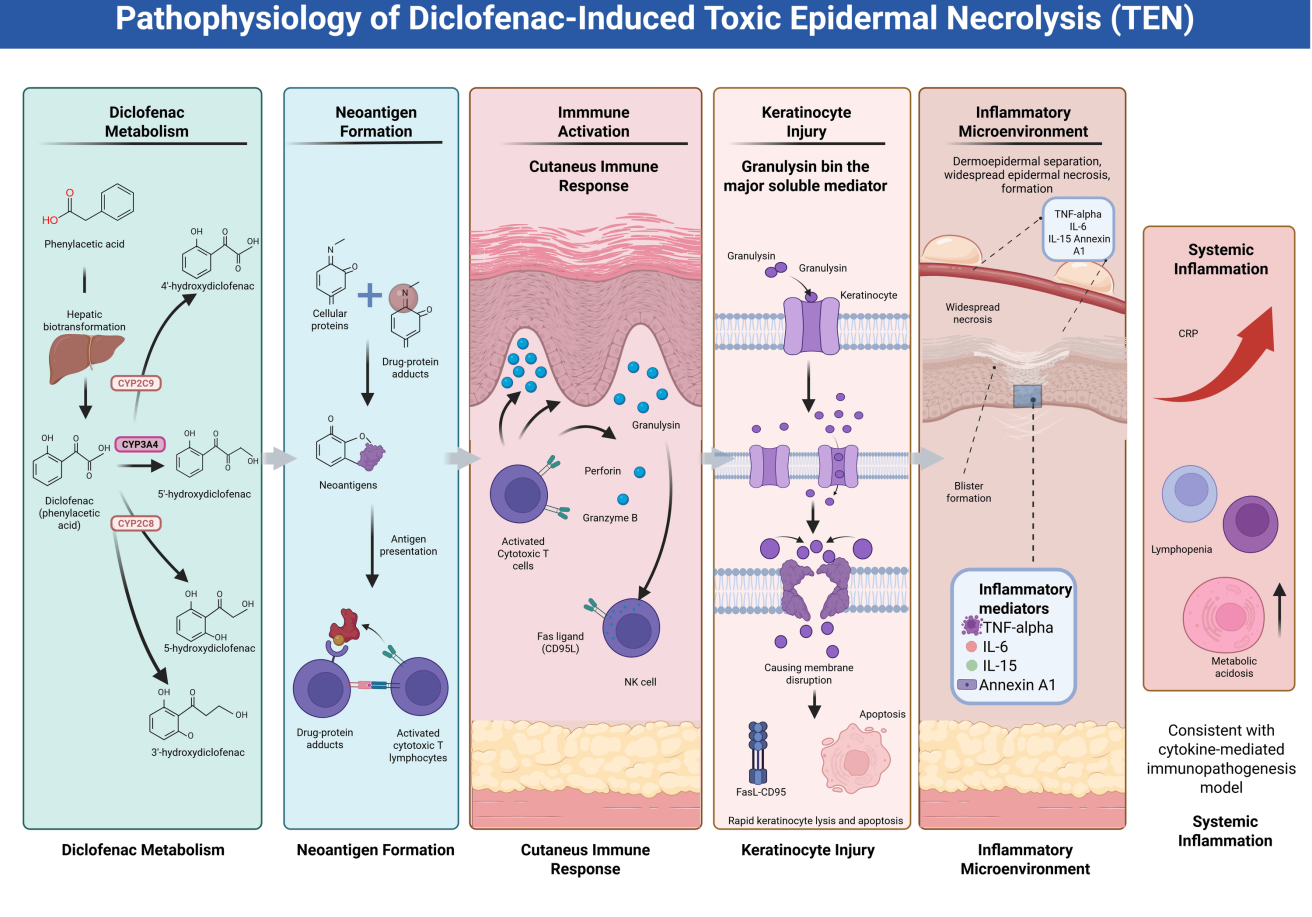
Pathophysiology of diclofenac-induced TEN CD95: cluster of differentiation 95; FasL-CD95L: Fas ligand-CD95 ligand; CRP: C-reactive protein; CYP: cytochrome P450; CYP2C9: cytochrome P450 2C9; CYP3A4: cytochrome P450 3A4; CYP2C8: cytochrome P450 2C8; IL: interleukin; IL-6: interleukin 6; IL-15: interleukin 15; NK cell: natural killer cell; TEN: toxic epidermal necrolysis; TNF-α: tumor necrosis factor alpha The image was created manually by Natalia Quintero using BioRender integrated tools and available figures in the library (https://BioRender.com/nohk27x). No AI-generated BioRender features were used.

This report describes an unusual, severe presentation of fatal drug-related TEN documented in the modern literature, with the time between the initial exposure to the drugs and death being only four days, both occurring on the same calendar day as admission to the hospital. An evaluation of the case needs to be conducted from several perspectives, including the pathophysiology of TEN, its toxicological rationale in terms of diclofenac-induced skin reactions, causality evaluation, and its significance for pharmacovigilance practice. TEN represents the extensive end of the SJS-TEN continuum; this condition is a family of severe skin reactions that are caused by an immune system-mediated response to an injury in the epidermis and which leads to widespread keratinocyte apoptosis and ultimately dermoepidermal separation and epidermal necrolysis [1,2]. The primary mechanism underlying TEN's development involves the interaction of drugs/metabolites with major histocompatibility complex (MHC) class I molecules on antigen-presenting cells for processing and presentation to cytotoxic cluster of differentiation 8-positive (CD8+) T cells. In addition, activation of natural killer cells and natural killer T (NKT) cells contributes to the killing of keratinocytes [3,7], although it has been established that while Fas ligand-Fas receptor interaction was previously believed to be central to the process of keratinocyte apoptosis, granulysin is presently considered the major soluble effector molecule for causing cell death. Blister fluid concentrations of granulysin have been found to exceed levels of perforin, granzyme B, and Fas ligand [7-9]. Diclofenac is primarily metabolized by CYP2C9 in the liver but also has smaller roles from other hepatic enzymes, specifically CYP2C8 and CYP3A4, to produce the primary phase I metabolite of diclofenac, 4'-hydroxydiclofenac [10], which could be further oxidized to generate reactive quinone imines. The latter could bind to proteins, forming drug-protein adducts that may have significant immunological implications [11-13]. Severe cutaneous adverse reactions have been associated with genetic predispositions to these conditions, with established associations including abacavir, carbamazepine, and allopurinol; however, there are no established associations between genetic variations and NSAID- or diclofenac-induced TEN [7,14]. Systemic inflammatory response, indicated by high C-reactive protein (CRP) concentration (exceeding 320 mg/L), lymphopenia, and metabolic acidosis, is entirely compatible with the cytokine-mediated immunopathogenesis model (Figure 3).

Formal causality analysis was undertaken employing the Naranjo Adverse Drug Reaction Probability Scale and World Health Organization and Uppsala Monitoring Centre (WHO-UMC) criteria. According to the results, the overall score for the Naranjo algorithm was 2, which places it in the "possible" (scores 1-4) category. Two factors contributed to the score: a clearly established temporal association between the administration of diclofenac on day 2 and the appearance of symptoms on day 3 (+2) and confirmation by clinical signs, including a positive Nikolsky sign, violaceous macules, and bullous erosions (+1). One point was deducted, as there were potential other causes from among pharmacological agents, i.e., febuxostat, etoricoxib, and pregabalin, which are known to cause serious skin reactions [5,15,16].

No additional points were accrued because diclofenac was a one-time exposure (thus, no chance to improve after discontinuation or rechallenge), plasma levels of the drug were unavailable, and there were no previous recorded reactions to diclofenac or any other related medication. In accordance with the WHO-UMC causality criteria, the relationship between diclofenac and TEN is "possible" as the drug was administered before the occurrence of symptoms (temporal association) and there is a pharmacologic basis to support an association. However, it is impossible to rule out other potential aetiologies entirely. In terms of toxicology, the development of mottled skin and blisters occurred within approximately 24 hours of diclofenac use. This timeline is possibly indicative of a type IV hypersensitivity reaction that had previously been generated in response to either previous use or previous sensitization to the drug, which may also explain why there was such a short time interval from drug administration until symptom onset.

As previously reported within the ALDEN framework for drug-induced epidermal necrolysis, those who develop drug-induced epidermal necrolysis typically experience significantly shorter latencies when they have been previously sensitized or previously exposed [6]. The number of case reports linking TEN-SJS to diclofenac intake is small, yet collectively suggests a potential causative link. Mockenhaupt et al.'s case-control analysis conducted in multiple countries found that NSAIDs are one of the drug classes with the highest relative risk for severe cutaneous reactions, particularly among oxicam derivatives regarding TEN and SJS [5]. In individual case reports, TEN was noted after exposure to diclofenac in oral, intramuscular, and suppository forms [6,17]. The clinical characteristics reported, such as involvement of the mucous membranes, presence of the Nikolsky sign, and inflammatory systemic activation, corresponded well with those observed. This case stands out from others due to its particularly severe course, characterized by extensive involvement of 40.5% of body surface, a high SCORTEN score of 5, multiple organ dysfunction at the time of admission, and death occurring shortly after admission, which emphasizes the vulnerability of elderly patients with comorbid conditions to the fulminant course of TEN [1,18].

From a clinical toxicological perspective, the management of suspected TEN due to medication use necessitates various immediate and simultaneous steps. The identification of the offending drug(s) and their immediate discontinuation represent the most significant interventions that have been proven to lead to favorable outcomes; the more days spent under exposure to the causative agents following the onset of symptoms, the higher the mortality rates [19]. As can be seen in this report, the potentially causative drugs were either given as a single dose or taken for the last time on the evening of day 4, before being admitted to the hospital on day 5. Other than the drug discontinuation, treatment of patients with TEN involves their prompt transportation to a specialized burn unit or intensive care unit, proper wound care, fluid/electrolyte resuscitation, nutrition support through enteral administration of food, ophthalmologic examination, and surveillance for possible infection [20]. Immunotherapy, both systemic and topical, is still highly controversial in TEN therapy, with conflicting evidence surrounding the practice; in this case, corticosteroids were used while cyclosporine was withheld owing to acute renal damage [3,9].

The fatal outcome seen in this patient is a result of the combination of several known risk factors, such as advanced, extensive body surface area impairment, severe metabolic acidosis, acute kidney injury, thrombocytopenia, and delay in presentation after multiple days of unknown illness. The score of 5 on the SCORTEN scale gave insight into the severity of the patient's condition, using a reliable risk prediction system at the time of admission. The quick development of multiorgan failure and respiratory and cardiac arrest due to hyperkalemia-related arrhythmia was the ultimate consequence of deranged skin barrier function, systemic inflammation-induced vasodilation, potassium retention in the kidneys, and metabolic acidosis, which is typical for fulminant TEN [1,18].

Pharmacovigilance concerns emerging from this case cannot go unmentioned. Diclofenac ranks as one of the most widely taken analgesic agents in the world, used in several forms both in hospitals and in communities. In its intramuscular form, which is regularly taken to manage acute pain, such as in cases of gouty flare-ups, it is fast-acting and provides high blood levels of the substance, thus potentially increasing antigenic load through systemic exposure when taken by a vulnerable individual. This particular case lends weight to the notion that TEN should be included as a very rare but known side effect of using diclofenac for pharmacovigilance purposes. Clinicians should remain aware of the possibility of rare but severe cutaneous adverse reactions associated with diclofenac and other NSAIDs, particularly in elderly patients with polypharmacy and recent drug re-exposure.

Limitations

This case report has many limitations. The first is that the diagnosis of TEN was made based on clinical evidence and, therefore, without histologic evidence. A skin biopsy could not be performed because the patient's condition was so serious and rapidly deteriorating. Because of this limitation in making a definitive diagnosis, it would have been impossible to determine if the case was an example of TEN versus some other severe form of necrosis/purpura/bullae. The second limitation is that the rapid progression of this patient's symptoms meant that he did not have an opportunity to undergo comprehensive testing to rule out other possibilities. For example, conditions like purpura fulminans, disseminated intravascular coagulation (DIC), toxic shock syndrome, necrotizing fasciitis, meningococcal sepsis, thrombotic vasculopathy (TV), and sepsis-associated skin failure (SASF) can all appear similar to TEN. While the patient had laboratory abnormalities, including shock, thrombocytopenia, coagulopathy, renal dysfunction, metabolic acidosis, numerous erosions, and a positive Nikolsky test, there are no records to document a thorough targeted investigation of these diseases. Finally, while it appears that the temporal relationship between the initiation of diclofenac and the development of this patient's symptoms is consistent with a drug-induced reaction, the toxicology review was unable to confirm that diclofenac caused the symptoms. Many recently reinitiated drugs potentially could have contributed to his reactions.

## Conclusions

In this case, the patient experienced fatal and fulminant TEN after being exposed to several new medications as well as one 75 mg dose injection of diclofenac. The SCORTEN score was 5, and the total body surface area affected by the reaction was 40.5%. Thus, this case is an example of how severe TEN can be in geriatric populations that are also affected by comorbidity and polypharmacology. As such, from a toxicological standpoint, this case represents a possible idiosyncratic or immune-mediated severe cutaneous adverse drug reaction where drug-related T-cell activation is responsible for widespread keratinocyte destruction. A score of 2 (possible) was obtained on a causality assessment utilizing the Naranjo algorithm and thus supports that the reaction could have been caused by one or more of the many drugs the patient recently began taking. Therefore, this case demonstrates the potential for rapid progression and death from TEN after the patient has recently taken numerous medications, including diclofenac. Prompt removal of potentially offending medications along with supportive critical care remains essential in improving outcomes.
